# Investigating the relationship between online information seeking and translation performance among translation students: The mediating role of translation self-efficacy

**DOI:** 10.3389/fpsyg.2022.944265

**Published:** 2022-09-23

**Authors:** Sha Lu, Wang Xiangling, Ma Shuya

**Affiliations:** School of Foreign Languages, Hunan University, Changsha, China

**Keywords:** online information seeking, translation performance, translation self-efficacy, mediation model, translation students

## Abstract

The widespread use of online information resources by translation students has motivated an increasing number of researchers to investigate the relationship between online information seeking and translation performance. However, these studies mainly address the direct effect of online information seeking on translation performance, thus failing to explore and identify the internal psychological mechanisms. This study, therefore, explores the mediating role of translation self-efficacy in the relationship between online information seeking and translation performance. A total of 314 translation students in China completed questionnaires on online information seeking and translation self-efficacy, and translation performance was measured by assessing the quality of a translation task given to them. Results showed that translation self-efficacy partially mediated the association between online information seeking and students’ translation performance. These findings can contribute to our understanding of the role that translation self-efficacy plays in information seeking behaviors and the emotional states of translation students in translator training.

## Introduction

Translator training has gained increasing attention in tertiary institutions and industrial markets since more quality translators are urgently needed in global communication (Tao, 2019). Translation performance, which refers to the degree to which a translator successfully accomplishes translation tasks, is a robust indicator for examining whether the students have achieved educational goals in translator training (Károly, 2012; Yang and Wang, 2020; Wang and Wang, 2021). A growing number of studies have indicated that translation performance could reflect students’ abilities to use translation strategies to solve problems (Kiraly, 2014; Hubscher-Davidson, 2017; Yang and Wang, 2020) and the extent to which students engaged in completing translation tasks (Rojo and Caro, 2016; Ge, 2019; Lin et al., 2021).

In translation studies, online information seeking has been found to be significantly correlated with translation process (e.g., Hvelplund, 2019; Sycz-Opoń, 2019; Prieto Ramos, 2020). Previous studies have shown that online resources such as search engines, electronic dictionaries, and specific portals, as compared with seeking information from print resources, enable translators to acquire better information in a more efficient way when they experience uncertainty in the problem-solving process (e.g., Hvelplund, 2017; Kuznik and Olalla-soler, 2018; Whyatt et al., 2021). However, mixed findings were reported on the impact of online information seeking on translation performance. On the one hand, online information resources are viewed as powerful tools that help translators gain helpful information effectively (e.g., Man et al., 2019; Sycz-Opoń, 2021), which might positively impact translation performance. On the other hand, recent studies indicated no direct relationship between the variety of online resources and final translation quality because of an inefficient and repetitive information seeking process, during which students selected improper resources without considering their information needs and the quality of information (Olalla-Soler, 2018; Cui and Zheng, 2020). In other words, the effect of online resources can be mitigated by individual factors such as the inadequacy in selecting and assessing information resources.

Prior research has proved the critical role of self-efficacy in influencing users’ performance (e.g., Kuhlthau, 1991; Wilson, 2000; Flavian-Blanco et al., 2011; Lopatovska, 2014; Jamali and Shahbaztabar, 2017). Research on this topic has focused on how it influences users’ behaviors to motivate themselves, organize search strategies, and increase satisfaction (e.g., Bronstein and Tzivian, 2013; Yan et al., 2017; Alshahrani and Pennington, 2019). In this study, we focus on translation self-efficacy as the factor that might have an influence on online information seeking and translation performance for the following reasons. Firstly, self-efficacy, derived from Bandura’s social cognitive theory, refers to the strength of an individual’s confidence in the ability to accomplish tasks (Bandura, 1993). Previous research has proved that self-efficacy before performing search activities can affect the motivation for accomplishing the tasks and thus affect the final task performance (Ross et al., 2016). Secondly, translation is considered as a complex and high-cognitive activity affected by different types of factors such as translation self-efficacy (Rojo and Caro, 2018; Ghobadi et al., 2021). Therefore, translation self-efficacy during online information seeking can influence the allocation of cognitive resources and the amount of effort and perseverance to be applied to perform tasks, especially in the face of difficulties (Bolaños-Medina, 2014; Bolaños-Medina and Núñez, 2018). Finally, translation self-efficacy entails self-reflection of one’s competence and will affect the development of translators’ information literacy (Pinto and Sales, 2008; Massey and Ehrensberger-Dow, 2011; Haro-Soler and Kiraly, 2019). In this regard, this study takes translation students as the participants, aiming to provide deep insights to investigate how to improve students’ translation performance and their information literacy.

Previous research has shown that online information seeking may affect translation performance (e.g., Hvelplund, 2019; Sycz-Opoń, 2019; Prieto Ramos, 2020). However, most of these studies have mainly concentrated on exploring the direct relationship between the two variables. Little attention has been paid to the study of individual factors such as translation self-efficacy and how it influences the relationship between online information seeking and translation performance. Hence, this study aims to extend the current research to investigate the internal psychological mechanism that underlies the association between online information seeking and translation performance, specifically through exploring the role of translation self-efficacy in this relationship. On the basis of previous research, this study uses online information seeking as the independent variable, translation self-efficacy as the mediating variable, and translation performance as the dependent variable to explore the possible associations between online information seeking, translation performance, and translation self-efficacy.

## Literature review and hypothesis development

### Online information seeking and translation performance

Translation is a complex decision-making activity during which translators encounter different types of problems and employ a set of strategies to deal with them (Enríquez Raído, 2014). According to PACTE (2005), translators need both internal and external support for solving problems, and the latter refers to information resources. The Internet has changed the way translators consult information, shifting from traditional resources such as printed dictionaries to digital forms. With the Internet and smart devices, translators can get easy access to a huge range of online resources that help them to gain useful information effectively and efficiently (Daems et al., 2016; Whyatt et al., 2021).

Online information seeking has been found to be closely related to the translation problem-solving process (Araghian et al., 2018; Shih, 2019; Sycz-Opoń, 2021). Previous research has shown that the access to online information resources has become an indispensable part of working processes, especially for the young generation of translators, as it can provide abundant information to fulfil different types of translation tasks and assist translators in both comprehending and reformulating texts (e.g., Kuznik and Olalla-Soler, 2018; Hvelplund, 2019). In this sense, the process of online information seeking, i.e., locating, comparing, and assessing search results, facilitates the solution of translation problems and thus has a significant impact on translation performance. Furthermore, researchers have demonstrated that online information seeking can enhance students’ performance by promoting learning and knowledge acquisition. Based on the constructivist approach to learning, Rieh et al. (2016) proposed that online information seeking could be conceptualized as a learning process in which people analyze, evaluate, and use relevant information to create new knowledge through various search activities. It has been found that online information seeking had a significant positive effect on learning outcomes and knowledge acquisition (Chyr et al., 2017; Wu et al., 2018; Weber et al., 2019; Miraj et al., 2021), and therefore it could enhance translation performance through the learning elements. Based on the above discussion, this research proposes the following hypothesis:

*H1*: Online information seeking is positively associated with translation performance.

### Online information seeking and translation self-efficacy

Previous studies have shown that some personal factors can be linked with translators’ online information seeking. For instance, Zheng (2012) found that professional translators tended to use a wider range of consultation sources and showed an investigative and diligent attitude towards their consultation behaviors, as compared with novices and semi-professional translators. Enríquez Raído (2014) further analyzed the relationship between subjective factors (translation expertise, information searching skills, and domain knowledge) and information seeking behaviors. It was found that students tended to carry out shallow searching strategies due to a lack of web search expertise. Moreover, Shih (2019) exposed that translation students should pay attention to managing affective factors such as frustration and mental fatigue in their processes of information seeking since these factors might harm the final quality. These findings indicate that translators’ information seeking behaviors can be influenced by some personal factors such as translation experience and skills. Little is known about the effect of emotional factors such as translation self-efficacy on online information seeking.

In the field of translation studies, translation self-efficacy is considered to be an essential element in the translation competence model. For instance, PACTE (2005) translation competence model comprises five sub-competencies (bilingual, extralinguistic, knowledge of translation, instrumental and strategic sub-competence) and psycho-physiological components, including translation self-efficacy. Since these competencies are interacted with and integrated into every translation act, translation self-efficacy may have an influence on the development of translation competence. In addition, Haro-Soler and Kiraly (2019) defined translation self-efficacy as “a translator or translation student’s confidence in their ability to translate the text at hand under the specific conditions and situational constraints stated in and implied by the brief or assignment.” (p, 261). On this basis, we defined translation self-efficacy as the belief in the ability to accomplish translation tasks to solve translation problems.

Established within the frame of social cognitive theory, translation self-efficacy is based on four sources: mastery experience, vicarious experience, verbal persuasion, and physiological states. Among these four sources, mastery experience, referring to previous task experiences or training, is the most prominent source of self-efficacy (Alshahrani and Pennington, 2019). Bandura (1991) argued that the repeated successful experiences could be valuable in fostering more positive expectations and offering reliable guides for improving self-efficacy. Also, Bandura (1993) argued that people developed their self-efficacy not only through their own experience but from observing the behaviors and results of others. Through this observation, individuals could learn about how to organize new behavioral patterns, especially in difficult situations (Bandura, 1997).

Thus, students’ online information seeking may have an impact on their translation self-efficacy. On the one hand, online information resources play an important role in enriching students’ mastery experiences during the translation process and help to improve translation self-efficacy (Bolaños-Medina and Núñez, 2018). On the other hand, online information resources such as social networking sites provide opportunities for students to communicate and share their knowledge (Hvelplund, 2017). For example, students could pose questions and obtain feedback from professionals without travelling long distances (Alshahrani and Pennington, 2019). In addition, students could also share their experience of translating and discuss scientific problems with others through different information channels (Shih, 2019; Sycz-Opoń, 2019). Such interactive process can lead to vicarious experience, which may, in turn, improve their translation self-efficacy. Therefore, it is reasonable to postulate a linkage between online information seeking and translation self-efficacy. The second proposed hypothesis is as follows:

*H2*: Online information seeking is positively associated with translation self-efficacy.

### Translation self-efficacy and translation performance

According to Bandura’s social cognitive theory, self-efficacy plays a significant role in the choices that people make, the amount of effort they put into certain tasks, how long they persevere through difficulties, and the degree of stress they experience. In educational settings, numerous empirical studies have shown the positive effect of self-efficacy on task performance (Yeo and Neal, 2006; Niemivirta and Tapola, 2007; Talsma et al., 2019). It appears that students with high self-efficacy exert more task-related effort and persist longer in the face of obstacles, which, in turn, enhances their chances of success and task performance (Khong et al., 2017). In contrast, individuals with low self-efficacy tend to have low aspirations to pursue their goals and fail to complete tasks (Clark, 2017). Additionally, self-efficacy has also been found to influence students’ cognitive process through increased use of deep processing strategies and metacognitive strategies, and then, in turn, influence one’s performance on tasks (Themanson and Rosen, 2015).

Translation performance, also known as translators’ performance in completing translation tasks, has been shown in empirical research to be easily influenced by emotional factors such as translation self-efficacy (Rojo and Caro, 2016; Hubscher-Davidson, 2017; Lehr, 2021). Specifically, Araghian et al. (2018) compared problem-solving strategies of translation students with high and low levels of translation self-efficacy. Results indicated that students with high self-efficacy tended to use more efficient strategies for solving translation problems. Compared with high self-efficacious students, students with low self-efficacy showed lower translation efficiency in performing translation tasks due to repeated attempts at text production and over revision, which ultimately affected their translation performance. Moreover, Bolaños-Medina and Núñez (2018) found that individuals with a high level of translation self-efficacy performed better in source language reading comprehension, tolerance of ambiguity, and the searching process for documentary information. Therefore, based on social cognitive theory and previous studies, we proposed Hypothesis 3 as follows:

*H3*: Translation self-efficacy positively predicts translation performance among translation students.

### The mediating role of translation self-efficacy

This study hypothesizes that translation self-efficacy mediates the relationship between online information seeking and students’ translation performance. The rationale for the mediation hypothesis is based on the information seeking models. Kuhlthau (1991) proposed an information search process model that emphasized the role of emotions in the interaction between humans and machines at every stage of the search process. He argued that the user’s thoughts, feelings, and information seeking behaviors interacted with each other and ultimately influenced the search performance (Kuhlthau, 1991). The model of information behavior proposed by Wilson (2000) combines social learning theory and other theories, and it focuses mainly on the context of information needs, activation mechanisms, intervening variables, and information seeking behaviors. In his model, psychological factors such as self-efficacy are one of the essential mediating variables that affect users’ information needs and final search performance. He then hypothesized that one of the motives for information seeking was to acquire information to improve one’s self-efficacy in dealing with problems (Wilson, 1997). In addition, Bandura (1997) explained that self-efficacy could also play a role in mediating the effect of skills on subsequent performance by influencing the level of effort and persistence when faced with obstacles.

Further, evidence from empirical studies also provides support for the role of self-efficacy as a mediator of online information seeking and users’ performance. Zhu et al. (2011) studied the mediating and moderating roles of academic self-efficacy in information seeking and showed that academic self-efficacy would be generated during the information seeking process, which ultimately regulated the information selection behaviors by the interaction of actions and cognition. In the same line, the study by Yu et al. (2014) indicated that the use of online information resources was critical to maintaining and improving students’ self-efficacy, which could further contribute to their performance. Therefore, we propose the fourth research hypothesis:

*H4*: Translation self-efficacy plays a mediating role in the association between online information seeking and translation performance.

### The current study

**Figure 1:**
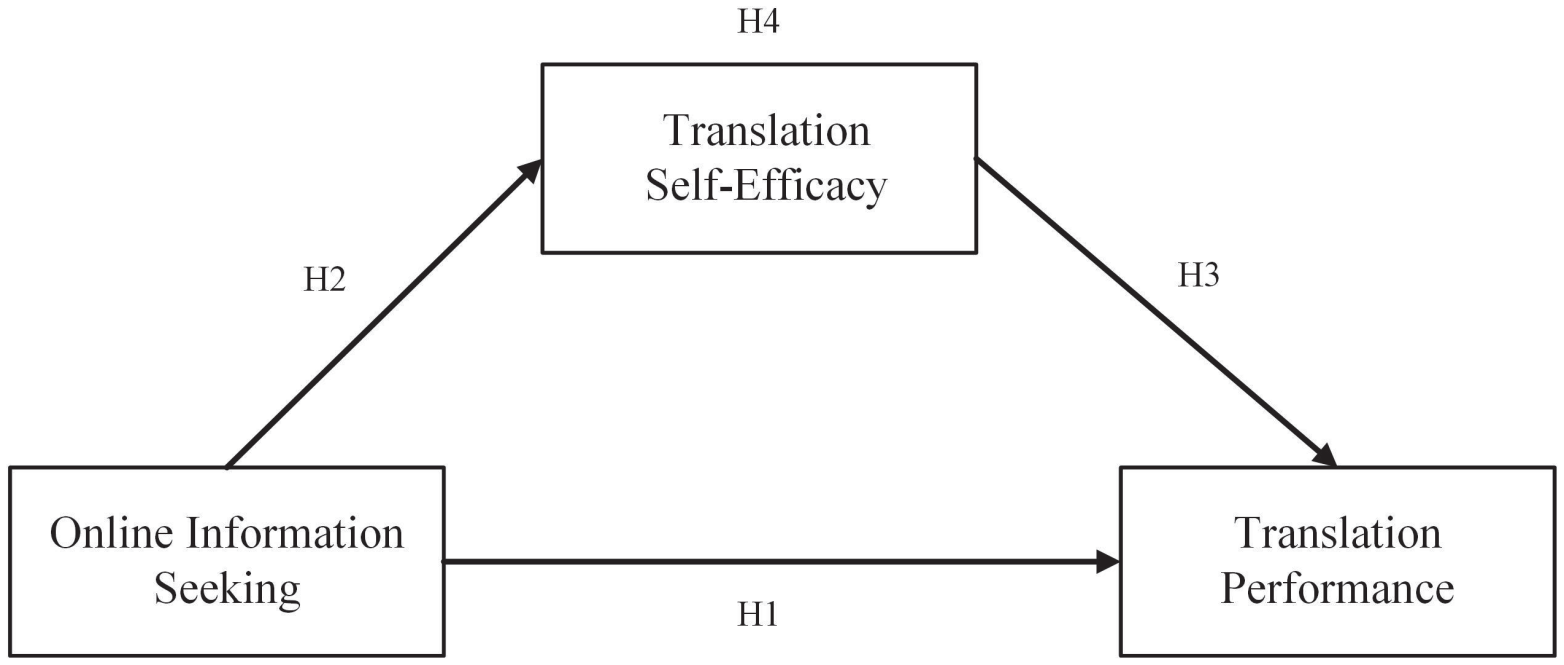
Conceptual framework.

Based on previous studies, we develop the conceptual framework in Figure 1. First, the conceptual framework proposes a positive relationship between online information seeking and translation performance; Second, it proposes a positive association between online information seeking and translation self-efficacy; Third, it proposes a positive relationship between translation self-efficacy and translation performance; and finally, it proposes a positive indirect association between online information seeking and translation performance as mediated by translation self-efficacy.

## Materials and methods

### Participants

Based on convenience sampling, we selected 320 undergraduate and post-graduate students majoring in translation studies as the respondents. Data from 314 students were used for the analyses, as 6 responses were excluded due to incomplete information. The population was made up of 239 females and 75 males, with an average age of 22.49 years (range 20–27, *SD* = 1.36 years). All participants had the same language background, with Chinese as their first language and English as their second language. Since previous studies have demonstrated that translation experience and language proficiency could affect students’ consultation performance (e.g., Zheng, 2012; Enríquez Raído, 2014), these two variables were used as the control variables. Therefore, we classified participants into two categories, namely novice translators and semi-professional translators, based on the translation experience they reported in the background questionnaire. Meanwhile, following Zheng (2012)‘s study, the scores participants obtained on the Test for English Majors-Band 8 (TEM-8) were also adopted as the criterion since it is one of the most important English tests for English majors in China. As a result, the group of novice translators consisted of 209 participants, and they scored between 60 and 70 points in the TEM-8 test with no part-time translation experience. The group of semi-professional translators consisted of 105 participants, and they scored over 70 points in the TEM-8 test with at least 3 years of part-time translation experience. In addition, the LexTALE test was used to measure the level of proficiency in English, which was adopted as another control variable in this study. Results showed that all participants scored more than 60 points on the LexTALE test, indicating that they were proficient in English (Lemhöfer and Broersma, 2012). Furthermore, all participants have reported that they were familiar with basic online information seeking techniques. After the experiment, they received a ¥40 reward for their work. The experiment was approved by the Ethics Committee of the College of Foreign Languages at Hunan University.

### Measurements

To address the key research objective, survey instruments were designed to measure online information seeking and translation self-efficacy level of participants. Then rubric analysis was adopted to measure translation performance.

#### Measuring online information seeking

Online information seeking was measured by Online Information Seeking Scale (OISS) with 12 items based on Choo et al. (1999) (see Appendix I). Respondents answered on a five-point scale ranging from “never” (1 point) to “always” (5 points), with higher scores reflecting more effective information seeking behaviors. Most of the items were modified to reflect students’ online information seeking process when performing translation tasks. The questionnaire was translated into Chinese by a professional translator with more than 5 years of translation experience. To verify the comprehensibility of the questionnaire, two translation teachers with more than 10 years of professional experience were invited to check the readability of the items. Further, a pilot study was conducted with 120 participants, and the pilot informants were excluded from the formal experiment. The Cronbach’s α of the 12-item scale is 0.861, indicating that OISS had satisfactory reliability (Hair et al., 2006). The assessment of major indices showed that the construct validity of OISS in this study was high for measurement (*χ*^2^/*df* = 1.477, RMSEA = 0.063, GFI = 0.907, AGFI = 0.858, CFI = 0.965, IFI = 0.966, TLI = 0.955), suggesting its validity for assessing participants’ online information seeking.

#### Measuring translation self-efficacy

For measuring translation self-efficacy, a questionnaire designed by Bolaños-Medina and Núñez (2018) was adopted with minor modifications in order to fit the purpose of the present study. It consisted of 20 items using a five-point Likert scale, scoring from 1 point (strongly disagree) to 5 points (strongly agree), with higher scores reflecting a higher level of translation self-efficacy. Similarly, the questionnaire was also translated into Chinese by a professional translator with more than 5 years of translation experience and the clarity of the items was checked by two translation teachers with more than 10 years of translation experience. Additionally, this questionnaire was piloted with a small number of people (106 students in total) to check its reliability and validity. The pilot informants were excluded from the formal experiment. Results showed that the scale had high internal consistency, with a Cronbach’s α of 0.891 (Hair et al., 2006). The assessment of major indices showed that the construct validity of the translation self-efficacy scale in this study was high for measurement (*χ*^2^/*df* = 1.134, RMSEA = 0.036, GFI = 0.892, AGFI = 0.850, CFI = 0.985, IFI = 0.985, TLI = 0.982), indicating that this questionnaire was valid for assessing participants’ translation self-efficacy.

#### Measuring translation performance

Translation performance was assessed based on the rubric designed by Hurtado Albir (2015), which is widely used to evaluate student’ translation performance in the context of translator training (see Appendix III). The rubric consists of two parts: quantitative and qualitative assessment. The quantitative assessment focused on the quality of translation products, while the qualitative assessment was used to assess the quality of the reports submitted by participants regarding their reflections on their translation process and presentation. In this study, two professional translators with more than 5 years of translation experience were recruited as assessors, who gave ratings for each item with a total score of 100. The average scores from the two assessors were calculated to measure the translation performance.

### Material

In this study, an English text from a patent document was selected as the source text (ST), and there were no Chinese translations available online (see Appendix IV). Before the formal experiment, we conducted the readability test to make a holistic assessment of the ST. As shown in Table 1, several quantitative indicators showed that a reader would need to complete 12 years of schooling to comprehend the text. Meanwhile, following Cui and Zheng (2020), we recruited 5 translation trainers to rate the translation difficulty of the text from 1 as “very easy” to 5 as “very difficult.” The mean value of translation difficulty for the text was 4.08, indicating the ST was appropriate for senior translation students to complete.

**Table 1:** Quantitative profiling of ST.

Indicators	ST
Length (in # of words)	386
Number of sentences	27
U.S. grade level	12
Flesch Kincaid Reading Ease	39.8
Gunning Fog score	15
SMOG index	10.9
Coleman Liau index	13.8
Automated readability index	9.7
Lix	52 (difficult)
Lexile measures	1210L–1400L

### Procedure

The study took place in the multimedia classroom from November 2021 to January 2022. Participants were asked to use their own laptops to finish the translation task as they normally work. The whole experiment could be roughly divided into two sessions. The first session started with a background information questionnaire, which collected their basic information and TEM-8 scores. Then, all students were instructed to complete a LexTALE test. They were informed that all data would be confidential and used only for research purposes.

In the second session, participants were required to complete an English–Chinese translation task. A 400-word English source text was sent to them in the form of an electric document *via* QQ group chat (a social software with functions of file transferring). Then, participants were provided with the corresponding translation brief, in which they were asked to translate the English text for a local scientific and technical services company. They were not allowed to use computer-assisted translation tools (e.g., translation memory and terminology management system) and machine translation to translate the whole source text. After translating the text, participants were asked to complete two questionnaires (Online Information Seeking Scale and Translation Self-efficacy Scale). The entire session for each participant lasts approximately 90 min.

### Data analysis

In this study, all data were analyzed using version 26 of the Statistical Package for the Social Sciences (SPSS) and PROCESS macro 3.3 (Hayes, 2013). First, descriptive statistical analysis and correlation analysis of the data were calculated using SPSS. Second, in order to test the hypotheses, Hayes’ PROCESS macro, which is a widely used procedure in humanities for testing mediation effects, was implemented on SPSS (Tafesse, 2020). In this study, 5,000 Bootstrapping was conducted to examine the indirect effects in the proposed hypotheses model (see Figure 1), and 95% CI’s exclusion of zero was taken as the criteria for the significance of the indirect effects (Hayes, 2013).

## Results

### Descriptive statistics and correlation analysis

Mean (*M*), standard deviation (*SD*), and correlations for the main variables in this study were reported in Table 2. For translation performance, the inter-rater reliability between the two assessors was calculated by Krippendorff’s alpha coefficient. Results showed that the coefficient was 0.810, suggesting that inter-rater reliability was satisfactory in the present study (Krippendorff, 2004). As shown in Table 2, the variables examined in the study had significant positive relationships. Online information seeking was significantly and positively correlated with translation self-efficacy (*r* = 0. 743, *p* < 0.01) and translation performance (*r* = 0.568, *p* < 0.01). Meanwhile, translation self-efficacy was significantly associated with translation performance (*r* = 0.640, *p* < 0.01). In other words, participants with a higher level of translation self-efficacy perform better on translation tasks.

**Table 2:** Descriptive statistics and correlations for the study variables.

Variables	*M*	*SD*	1	2	3
1. Online information seeking	44.653	9.414	1		
2. Translation self-efficacy	52.911	10.831	0.743^**^	1	
3. Translation performance	72.911	7.326	0.568^**^	0.640^**^	1

*N* = 314; *M*, Mean; *SD*, Standard deviation.

** *p* < 0.01.

### Hypotheses testing

In the current conceptual framework, online information seeking is used as the independent variable, translation performance as the dependent variable, and translation self-efficacy as the mediating variable. To control for the effects of language proficiency and translation experience, these two variables were entered into the model as covariates. The results of the model estimation are shown in Tables 3, 4.

**Table 3:** Mediation effects of translation self-efficacy on the relationship between online information seeking and translation performance.

	Model 1 (translation performance)		Model 2 (translation self-efficacy)		Model 3 (translation performance)	
	*β*	*t*	*β*	*t*	*β*	*t*
Translation experience (semi-professional = 1; novice = 0)	−0.085	−1.743	−0.005	−0.140	−0.082	−1.846
Language proficiency	−0.006	−0.133	0.069	1.785	−0.042	−0.944
OISS	0.571	12.301^**^	0.759	20.635^**^	0.177	2.714
TSE					0.519	7.927^**^
R^2^	0.332		0.580		0.445	
F	51.460^**^		142.984^**^		62.004^**^	

Each column is a regression model that predicts the criterion at the top of the column. Covariates: Translation experience; Language proficiency. OISS, online information seeking; TSE, translation self-efficacy.

** *p* < 0.01.

**Table 4:** The bootstrapping analysis of the mediating effects.

	Effect	Boot SE	Boot CI lower	Boot CI upper	Proportion (%)
Total effect	0.444	0.033	0.379	0.510	100
Direct effect	0.137	0.061	0.021	0.261	30.86
Indirect effect	0.307	0.050	0.211	0.406	69.14

*Hypothesis 1* posited the positive influence of students’ online information seeking on their translation performance. As presented in Table 3, in Model 1, after controlling for language proficiency and translation experience, students’ online information seeking was positively associated with their translation performance (*β* = 0.571, *p* < 0.01). Hence, H1 was supported.

*Hypothesis 2* predicted a significant positive association between online information seeking and translation self-efficacy. In Model 2, results indicated that after controlling for language proficiency and translation experience, online information seeking was positively related to translation self-efficacy (*β* = 0.759, *p* < 0.01), which strongly supported H2.

*Hypothesis 3* predicted a significant positive association between translation self-efficacy and translation performance. The results showed that, in Model 3, translation self-efficacy was positively correlated to translation performance (*β* = 0.519, *p* < 0.01). In other words, participants with a higher level of translation self-efficacy performed better in translation tasks. Thus, H3 was also supported.

Finally, hypothesis 4 predicted that translation self-efficacy would mediate the relationship between students’ online information seeking and their translation performance. As shown in Table 4, after controlling for covariates, the bias-corrected percentile bootstrap method demonstrated that the indirect effect of online information seeking on translation performance through translation self-efficacy was significant, *ab* = 0.307, Boot *SE* = 0.05, 95% *CI* = (0.211, 0.406), indicating that the sense of translation self-efficacy partially mediated the relationship between online information seeking and translation performance. The mediation effect accounted for 69.14% of the total effect. Therefore, H4 was also supported.

In summary, all the hypotheses in the research model were supported. The results of the hypothesis test are summarized in Table 5.

**Table 5:** Summary of the hypothesis test.

Proposed hypothesis	Results
H1: Online information seeking is positively associated with translation performance	Support
H2: Online information seeking is positively associated with translation self-efficacy	Support
H3: Translation self-efficacy positively predicts translation performance among translation students	Support
H4: Translation self-efficacy plays a mediating role in the association between online information seeking and translation performance	Support

## Discussion

This study sets out to explore the role of translation self-efficacy in the relationship between online information seeking and translation performance. In this section, we first discuss the findings related to the research hypothesis, then focus on the implication and directions for future research.

According to the first finding of the study, there was a positive relationship between online information seeking and translation performance, which was consistent with previous studies that the online information seeking behavior of translators had a significant impact on the final quality of the translation products (e.g., Enríquez Raído, 2014; Shih, 2019; Sycz-Opoń, 2019; Cui and Zheng, 2020). Specifically, online information resources are regarded as accessible and efficient resources for translators (Xu and Wang, 2011). These resources could stock plenty of updated and real-time information across various fields (Gough, 2017; Hvelplund, 2017; Kuznik and Olalla-Soler, 2018). Therefore, when students encounter difficulties in the translation process, they can consult information with low effort and cost through different online information resources to solve translation problems. At the same time, the use of various search tools could also help student translators to make judgements on the reliability, accuracy and authority of information obtained from the Internet (Enríquez Raído, 2014; Olalla-Soler, 2018; Hvelplund, 2019), thus helping to improve their translation performance.

The second finding of this study also demonstrated that online information seeking was positively related to translation self-efficacy. This is in line with previous research findings that have examined the role of online information seeking in improving self-efficacy (Bronstein, 2014; Shen, 2018; Miraj et al., 2021). In the present study, online information seeking provides students with the knowledge and problem-solving solutions that can be easily used, which has a direct influence on developing mastery experience and eventually increase their self-efficacy level (Zhu et al., 2011). In this regard, if translation teachers provide more authentic translation projects for students to exercise their information skills, it will enhance their mastery experience and eventually increase their self-efficacy level (Haro-Soler and Kiraly, 2019). Moreover, students can also improve their translation skills through various online information resources (e.g., knowledge sharing on social platforms by peers), thus enhancing their confidence in their abilities to complete translation tasks.

The third finding of the study showed a significant positive relationship between translation self-efficacy and translation performance, which concurs with the results of past research on the role that translation self-efficacy played in the translation process (e.g., Araghian et al., 2018; Bolaños-Medina and Núñez, 2018). This finding may be explained by the assumption that students with a higher sense of self-efficacy are likely to be more motivated and devote more effort to the translation task, thereby improving the quality of the final product (Bolaños-Medina, 2014). Furthermore, previous research has demonstrated that stronger efficacy beliefs are associated with a better ability to effectively adjust to the dynamic changes during the translation process, using effective translation strategies for solving problems, and a higher level of task accomplishment (Araghian et al., 2018; Yang and Wang, 2020). Further, students with a high level of confidence in their translation ability tend to perceive stress or anxiety as an energizing facilitator of action, devoting more effort and attention to making revisions, which positively impacts their translation performance (Lee, 2017; Haro-Soler, 2018). In comparison, students with a low level of self-efficacy may experience more uncertainty and stress during the process (e.g., Bolaños-Medina, 2014; Araghian et al., 2018). As a result, such anxiety can, in turn, bring about additional strain to adjust their strategies and make more improvements in their translation products (Rojo and Caro, 2016; Prieto Ramos, 2020; Sycz-Opoń, 2021).

Moreover, the results of the present study showed that translation self-efficacy partially mediated the relationship between online information seeking and translation performance, suggesting that online information seeking can influence translation performance through translation self-efficacy. This finding corroborates previous research findings on the positive role of self-efficacy in the association between information seeking behaviors and task performance (e.g., Zhu et al., 2011; Zha et al., 2015; Miraj et al., 2021). According to social cognitive theory, self-efficacy is conceived as the outcome of weighing, integrating, and evaluating information about one’s capabilities, which, in turn, regulate the choices people make and the amount of effort towards accomplishing a given task (Bandura, 1997). Previous studies indicate that people with a high level of self-efficacy tend to devote more time and effort to monitoring the searching process, avoiding getting lost in the process and assessing search results, which consequently contributes to better performance and productivity (e.g., Bronstein and Tzivian, 2013; Gkorezis et al., 2017; Eldakar and Kenawy, 2020). In addition, the translation competence model proposed by PACTE identifies translation self-efficacy as one of the essential psychological states that can have a significant impact on instrumental competence and other sub-competencies (Hurtado Albir, 2017). Therefore, students who are more self-efficacious tend to have more persistence when facing challenges and failure during the search process and thus achieve better performance. However, those with doubts about their abilities might experience more negative thoughts about their achievements when searching online, and these negative reactions would, in turn, influence their final task accomplishment.

The present study explored the internal psychological mechanism and factors influencing translation performance. These findings have several significant implications. From a theoretical point of view, the present study extends Bandura’s self-efficacy theory to explore students’ online information seeking, contributing to the literature by proposing and testing a model in which translation self-efficacy partially mediated the relationship between online information seeking and students’ translation performance. From a practical perspective, this study highlights the importance of translation self-efficacy in enhancing students’ translation performances, which reminds translation teachers of the importance of translation self-efficacy when seeking to improve students’ information literacy. Haro-Soler (2019) argued that instructors and trainers could facilitate students’ translation self-efficacy by helping them become aware of their actual performances and the problems. To foster students’ self-awareness, some technical instruments can be integrated across the curricula. For example, screen recording is a useful diagnostic tool for students to reflect upon their own problems during learning activities. It can also increase their awareness of their translation processing styles (Angelone, 2019). E-portfolios could also monitor and examine achievements at different learning stages to facilitate self-efficacy levels (Rico, 2017). Further, curriculum developers could design progressive tasks from authentic translation projects to encourage students to practice their skills. This could help students enrich their mastery experience and improve their confidence in the ability to deal with complex problems (Kiraly, 2014). In addition, instructors and trainers could offer timely positive feedback and facilitate effective peer feedback for boosting translation self-efficacy.

Despite the contributions mentioned above, this study has some limitations. Firstly, the sample is limited to translation students in China. Future studies can use a broader sample of participants in various areas to generalize the research. Secondly, the translation task performed in this study was an English text from the second language (L2) into the first language (L1) without considering other language pairs or translation directionality. Thirdly, the translation performance was measured through one specific text type of translation. Thus, it would be worthwhile to explore further the relationship between translation self-efficacy and other types of translation texts, such as literary translation. In addition, future studies could explore other specific emotional elements, such as anxiety and tolerance of ambiguity, as they are essential to translators’ problem-solving process and performance.

## Conclusion

In summary, this study investigated the relationship between students’ online information seeking and translation performance and the mediating role of translation self-efficacy in this relationship. Results showed that both online information seeking and translation self-efficacy had an impact on translation performance. Furthermore, the mediation analysis showed that translation self-efficacy partially mediated the relationship between online information seeking and students’ translation performance. These findings suggest that translators’ online information seeking is a complex cognitive process that integrates emotional factors. For further research, researchers could explore other specific emotional elements such as anxiety and uncertainty during the information seeking process. In addition, translation teachers should consider students’ affective states when designing more practical teaching methods to promote information literacy.

## Data availability statement

The raw data supporting the conclusions of this article will be made available by the authors, without undue reservation.

## Ethics statement

The studies involving human participants were reviewed and approved by Hunan University. The patients/participants provided their written informed consent to participate in this study.

## Author contributions

SL conceived the original idea, carried out the experiment, and wrote the manuscript. WX contributed substantially to the writing and revising of the manuscript. MS provided critical feedback to the improvement of manuscript. All authors contributed to the article and approved the submitted version.

## Funding

This work was supported by Hunan Provincial Innovation Foundation for Postgraduate (grant number CX20210416).

## Conflict of interest

The authors declare that the research was conducted in the absence of any commercial or financial relationships that could be construed as a potential conflict of interest.

## Publisher’s note

All claims expressed in this article are solely those of the authors and do not necessarily represent those of their affiliated organizations, or those of the publisher, the editors and the reviewers. Any product that may be evaluated in this article, or claim that may be made by its manufacturer, is not guaranteed or endorsed by the publisher.
