# Supplementary material for: Investigating the relationship between online information seeking and translation performance among translation students: The mediating role of translation self-efficacy
Source: Front Psychol. 2022 Sep 23;13:944265. doi: 10.3389/fpsyg.2022.944265 (PMC9539674; doi:10.3389/fpsyg.2022.944265)
Supplement: Supplementary file 1 [file Data_Sheet_1.docx]

Supplementary Material

# Appendix Ⅰ: Online Information Seeking Scale (OISS)

1. When I think of translation problems, I go to the Internet to search for information.
2. I use electronic information resources that are related to specific subjects in the translation task.
3. I follow links on starting pages/websites to other content-related sites in order to solve translation problems.
4. When pointers or references from an initial resource are followed, I dig into the initial resource; in the reverse direction, I identify and follow up on other resources that refer to an initial resource or document.
5. I expose myself to a resource space by scanning top-level pages: lists, headings, site maps.
6. I perceive that the behavior (browsing) leads to outcomes such as serendipitous findings, modification of information needs, learning, enjoyment, and so on.
7. I select useful electronic information resources by bookmarking, printing, copying, and pasting, etc.
8. I choose/ start at differentiated, pre-selected electronic information resources of known content.
9. I validate electronic information resources updates using e.g., push notifications, or profiles.
10. I revisit “favorite” electronic information resources for new information related to the translation task.
11. I systematically work through a particular resource or resources in order to extract information interest at the site (a form of retrospective searching).
12. I directly consult the resource, or indirectly look through electronic information resources to extract the information I need.

# Appendix Ⅱ: Translation Self-efficacy Scale

1. I can identify the text genre of a given document.
2. I can analyze both source text production and target text reception communicative situations.
3. I can analyze the skopos or the main function which will be required for a given target text.
4. I can select proper translation tools related to the translation tasks.
5. I can identify translation problems (comprehension problems, production problems, combined problems).
6. I can generate different alternative solutions for translation problems.
7. I can evaluate different alternative solutions for translation problems.
8. I can make appropriate decision in order to solve translation problems.
9. I can use various kinds of online resources during translation process (direct translation / inverse translation).
10. I can control the time spent on searches at each stage (orientation, development, revision) of the translation process properly.
11. I can evaluate different types of online sources according to search needs.
12. I can find proper resources that could assist translation problem solving (direct translation / inverse translation).
13. When translating, I can solve most problems if I make the necessary effort.
14. I am confident that I can use various translation techniques in order to convey the meaning of source texts in the most acceptable form in the target text.
15. I understand how to translate better than I did before.
16. I can always learn from every translation assignment.

# Appendix Ⅲ: Assessment rubric for translation performance (Hurtado Albir, 2015)

| **Aspects to be assessed** | | **Score** |
| --- | --- | --- |
| Translation quality | Expression of the meaning of the original text   - Same information - Same clarity - Same register | 15 |
|  | Composition in the target language   - Conventions of written language (correct orthography and typography) - Vocabulary (appropriateness and richness) - Morphosyntax (good use of verb tenses and modes, prepositions, etc.) - Cohesion (good use of connectors and referential elements) - Coherence (ideas well organized and clearly presented) | 25 |
|  | Level of communication of the target text   - Overall quality of the target text - Appropriateness in terms of the genre’s conventions - Appropriateness in terms of the translation’s purpose and target audience | 15 |
| Report quality | Depth of reflection on the translation process   - Organization of work - Problems identified - Explanations of solutions - Alternative solutions - Documentary resources used - Reflection on the process (appraisal of the result, possible errors made, etc.) | 30 |
|  | Composition, structure, and presentation | 15 |

# Appendix Ⅳ. Material

A system is provided for analyzing a plurality of samples in a furnace. The system includes an upper holder including at least one opening adapted to engage at least one upper crucible. The system also includes a lower holder including at least one opening adapted to engage at least one lower crucible. The system includes a scale adapted to receive a lower crucible and weigh the lower crucible.

1. Field of the Invention

The present invention relates to crucibles, and in particular relates to stackable crucibles and systems for using stackable crucibles in furnaces.

1. Description of Prior Art

Thermogravimetric analyzers (TGA) are used to analyze moisture, volatiles and ash (coal and coke). Some systems have multi-sample capabilities and/or crucible covers for allow the analysis of several samples simultaneously. U.S. Pat. No.7,048,888 to Las Navas Garcia dis-cusses an automatic cover system for proximate analyzers and the like. An apparatus is provided to automatically cover and uncover crucibles according to a predetermined procedure in a proximate analyzer. A series of crucibles mounted in a first carousel is heated in a furnace. A second carousel mounted above the first carousel holds crucible covers. A mechanism synchronizes the movements of the carousels so that the heated crucibles are automatically covered and uncovered at the appropriate times during the analysis with a corresponding cover by lowering or raising the second carousel. The movements of both carousels are automatically controlled so that at appropriate points in the testing cycle they rotate simultaneously about a common central axis and a crucible is deposited on a weighing platform by controlling the vertical motion of the entire carousel apparatus. U.S. Pat. No.7,172,729 to Las Navas Garcia dis-cusses a mixed sample moisture or ash analyzer. In particular, an analyzer for moisture or ash testing is provided where a robotic arm retrieves a crucible and sample from a conveyor, inserts it into a small opening in the upper surface of the furnace chamber and deposits it in an aperture on a carousel located within the furnace chamber. The carousel in the furnace chamber manipulates the crucibles within the furnace chamber. The opening in the upper surface of the furnace chamber is positioned such that when the carousel is ready for loading or unloading, an aperture in the carousel for holding the crucibles is aligned with the opening.
